# Influence of Freeze-Dried Diet on Oral Hygiene Indicators in Strict Isolation Condition of an Analog Space Mission

**DOI:** 10.3390/ijerph19031367

**Published:** 2022-01-26

**Authors:** Barbara Janina Gronwald, Karina Kijak, Karolina Jezierska, Helena Anna Gronwald, Kamil Kosko, Mikołaj Matuszczak, Hanna Barbara Bielawska-Victorini, Wojciech Podraza, Leszek Orzechowski, Danuta Lietz-Kijak

**Affiliations:** 1 Doctoral Study at the Department of Propaedeutics, Physical Diagnostics and Dental Physiotherapy, Pomeranian Medical University in Szczecin, 71-210 Szczecin, Poland; 2 Scientific Student Group STO-MATER-FIZ at the Department of Propaedeutics, Physical Diagnostics and Dental Physiotherapy, Pomeranian Medical University in Szczecin, 70-111 Szczecin, Poland; karo0326@gmail.com (K.K.); mikolaj.matuszczak@gmail.com (M.M.); 3 Department of Medical Physics, Pomeranian Medical University in Szczecin, 71-073 Szczecin, Poland; karolina.jezierska@pum.edu.pl (K.J.); wojciech.podraza@pum.edu.pl (W.P.); 4 Department of Propaedeutics, Physical Diagnostics and Dental Physiotherapy, Pomeranian Medical University in Szczecin, 70-111 Szczecin, Poland; helenagronwald@gmail.com (H.A.G.); danuta.lietzkijak@gmail.com (D.L.-K.); 5 Individual Dental Practice Kamil Kosko, 62-510 Konin, Poland; kamil.kosko@gmail.com; 6 Department of Orthodontics, Pomeranian Medical University in Szczecin, 70-111 Szczecin, Poland; hania.bielawska@wp.pl; 7 LunAres Research Station, 64-930 Piła, Poland; orzechleszek@gmail.com

**Keywords:** analog space mission, freeze-dried diet, isolation, oral hygiene, oral health

## Abstract

Analog space missions were created to study the human factor in extraordinary conditions that would occur in future space habitats. Isolation has been shown to cause stress and disrupt individuals’ daily routine, which can also affect their oral hygiene and lead to an increased risk of dental caries and gingivitis. The astronauts’ specific freeze-dried diet is associated with “lazy” chewing, potential dehydration and vitamin A deficiency, which may adversely affect their saliva. The aim of this study is to investigate the influence of the freeze-dried diet on selected oral hygiene indicators in analog astronauts (AA) enduring strict isolation conditions during six consecutive analog space missions at the LunAres Research Station. During the experiment the oral hygiene and gingival inflammation status measurements were conducted on the group of AAs at the beginning and at the end of each mission. Measurements included four oral hygiene indicators: API, sOHI, PI by Silness and Loe and GBI by Ainamo and Bay. Each AA’s individual scores were noted and analyzed. Statistically significant reduction in the amount of plaque and intensity of gingival bleeding was observed over the course of the study, which could indicate positive results of applied oral hygiene procedures despite unfavorable dietary and stressful isolation conditions.

## 1. Introduction

Research on various aspects of life in space, conducted all over the world, is gaining momentum as the vision of conducting extra-terrestrial missions slowly becomes a reality. What a few decades ago seemed like a fantasy, is taken today with utmost seriousness, because we realize how much preparation and research still needs to be carried out. The main research center in the field of space exploration is the US National Aeronautics and Space Administration (NASA). However, the centers, particularly focusing on the human factor and its possible adaptation to the conditions of life in space, are primarily analog space bases where analog astronauts can face the difficulties and challenges of taking part in a mission in a controlled environment. Some of such research even provides insides into important team- and individual-level variables during stressful space analog events, while considering factors related to human psychology, which allows for a better understanding of the factors affecting astronaut teams in these contexts [1]. Other analog missions conducted in remote areas focus on preparing the analog astronauts and developing necessary laboratory analytical methods and techniques comparable with those that would likely be used in the future on Mars (AMADEE-18 mission; Dhofar area, Oman) [2].

**Figure 1:**
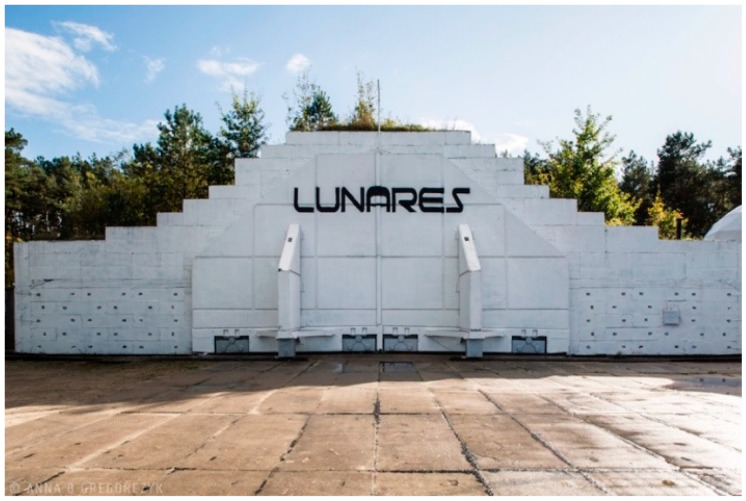
LunAres Research Station—Analog space base (Piła, Poland).

One of only six such centers in the world and the only one in Europe is the LunAres Research Station in Piła (Poland), established in 2017 (Figure 1).

**Figure 2:**
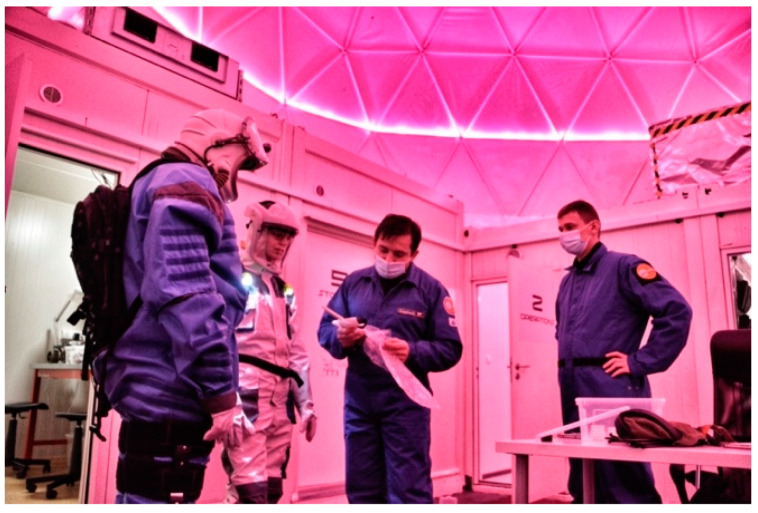
Analog astronauts during one of the analog space missions of Pandemic Isolation Campaign (2021).

This analog space base is housed in an extended and specially adapted airplane hangar, used mainly for research of a interdisciplinary nature, conducted during manned missions with the participation of scientists and collaborators from around the world (Figure 2).

The main goal of LunAres is to create a research platform to support scientific and technological development in manned space explorations, creating a unique research opportunity to conduct interdisciplinary experiments. The broad range of specialists are involved in the study from various fields, including extreme medicine, psychology, biotechnology, robotics and engineering, sustainability, extreme plant cultivation, sociology, and architecture. The possible observation and control of the indoor environment, as well as telemetry of the crew’s physical and psychological states allows us to observe the impact of simulated Martian and Lunar conditions on people staying periodically in strict isolation from the outside world, as well as to prepare procedures and solve problems that may arise during real space expeditions. This makes the center an indispensable link in preceding space research and cosmic expeditions—as demonstrated by Dr. Sian Proctor, crew member of Inspiration4, the first civilian flight into space (September 2021), who also trained at the LunAres Research Station.

**Figure 3:**
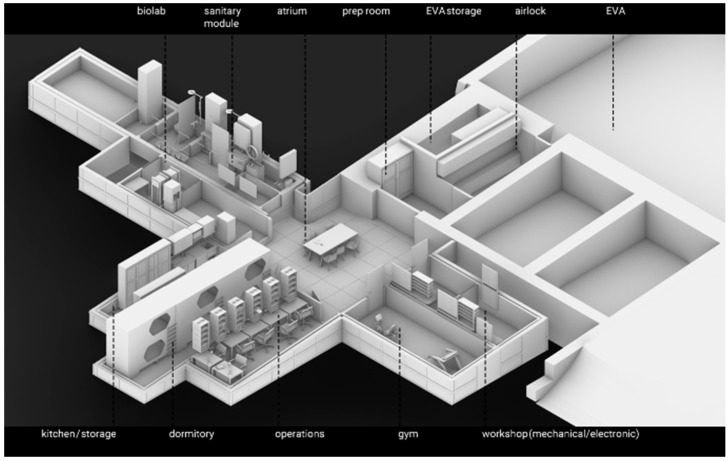
The infrastructure of the analogue space station in Piła.

The LunAres base is located at the post-military airport in Poland. The facility provides full isolation from the external environment including 250 square meters of EVA area (extravehicular activity area), allowing for 2 week missions for a 6-person crew. The infrastructure of the station provides constant monitoring of health and behavior of the crew (Figure 3).

Research on the influence of isolation and long-term stress on the physiology of the human body is extremely difficult due to the numerous variables that can determine the parameters measured. In order to eliminate the influence of accidental factors, the research team of the Department of Propaedeutics, Physical Diagnostics and Dental Physiotherapy of the Pomeranian Medical University in Szczecin has cooperated with LunAres Research Station since 2018. The conducted research provides an opportunity to examine the impact of various factors, e.g., freeze-dried diet, stress and isolation on the stomatognathic system. During the missions of the Pandemic Isolation Campaign, the emphasis was put on a freeze-dried diet and isolation.

It is important to consider some aspects of astronaut’s life—especially periods of strict isolation and high levels of stress. Scientists have demonstrated that isolation can induce stress and affect a person’s everyday routine. Any potential neglect of oral hygiene routine can occur, and, due to oral biofilm formation and development, impact a person’s health by increasing the risk of dental caries, periodontal disease (gingivitis or periodontitis) and peri-implantitis [3]. The oral cavity appears as an open ecosystem, with a dynamic balance between the entrance of microorganisms, colonization modalities and host defenses aimed to their removal; to avoid elimination, bacteria need to adhere to either hard dental surfaces or epithelial surfaces [3]. Potential plaque accumulations on tooth surface or gums (due to lack of proper oral hygiene) are ideal conditions for bacteria growth. Thanks to the wet and warm environment of the oral cavity with constant nourishment and access, bacteria in the oral plaque begin to develop into biofilms and maturate. Due to the maturation process, the biofilm colony produces more and more toxic by-products that stimulate the host’s immune responses. Studies closely examining inflammatory process occurring in affected periodontal tissues suggests the existence of an inflammatory response in these tissues by increased mRNA level of prostaglandin-endoperoxide synthase (PTGS2), multiple cytokines and chemokines, which arise mainly from resident fibroblasts, keratinocytes and endothelial cells [4]. As a result of the mentioned risks, all mission participants had to adhere strictly to the detailed daily schedule.

The freeze-drying process consists of drying food products after freezing them with the use of reduced pressure, resulting in the removal of 70% to 96% of the water. This procedure allows us to inhibit the microorganism’s growth and slow down enzymatic processes to very high degree [5,6]. This method ensures preservation of the product nutritional value, and because the food is dried, it is lighter and has a longer shelf life. These properties make a freeze-dried or lyophilized diet especially recommended for astronauts (but also climbers, sailors and soldiers) and in the 1960s, it was used for the first time on a larger scale for astronauts of the Skylab mission [7,8,9].

However, even most sophisticated food preservation methods are not ideal and during the free-drying process also occurs some loss of active ingredients, such as the phytochemical components of the products. It was proven that some tropical fruits can significantly lose their vitamin C content due to freeze-drying [10]. These deficits are impacted by the lyophilization process itself, e.g., the freeze-drying of the sea buckthorn berries at 20 °C shelf temperature and 30 m Torr vacuum pressure caused a 20% loss in vitamin C and in total carotenoids and a 35% loss in vitamin E, but only a 4% loss in total phenolics [11]. Also the type of bio-compound we try to preserve plays a big role in the potential loss of active ingredients of preserved food. Studies concluded that retention of vitamin C and phenolic content is best achieved by the process of freeze-drying, but in terms of the preservation of β-carotene, lycopene, vitamin E, unsaturated oils, and other lipid-based oxidizable bio-compounds, the lyophilization process could damage the quality of lipid-based bio-compounds, due to autocatalytic oxidative reactions accelerating at very low water activities, which are achieved during the freeze-drying procedure [12]. Nevertheless, when compared to other food preservation techniques, freeze-drying is usually a superior technology [13,14,15].

On the other hand, proper nutrition influences the course of developmental processes of individual elements of the masticatory organs and is responsible for maintaining the health of the tissues of the oral cavity [16,17]. For instance, a specific aspect of too-high food fragmentation in astronauts’ diet is associated with “lazy” chewing, potential dehydration or vitamin A deficiency, which may result in a reduction in the amount of saliva produced or its increased density [18]. In addition, dried food differs in consistency after rehydration from its regular state. Studies demonstrate that dried fruits after ingestion become “sticky” in the oral cavity and adhere to tooth surface, which can lead to plaque accumulation, local gingival inflammation or caries [19,20]. This only underscores the importance and need of research in this field.

The aim of the research was to investigate the influence of a freeze-dried diet on selected oral hygiene and gingival inflammation indicators in conditions of strict isolation during consecutive analog space missions in 2021 as part of the Pandemic Isolation Campaign at the LunAres Research Station.

## 2. Materials and Methods

Oral hygiene and gingival status studies were conducted in a group of 31 analog astronauts (AA) who consumed only freeze-dried products for 14 days and endured the rigorous conditions of isolation on the limited space of the analog habitat at the LunAres Research Station during 6 consecutive analog space missions in 2021 as part of the Pandemic Isolation Campaign.

5 AA (mission 1) + 6 AA (mission 2) + 6 AA (mission 3) + 5 AA (mission 4) + 4 AA (mission 5) + 5 AA (mission 6) = 31 AA

AAs participating in the study came from 15 different countries, thus, the study group was ethnically heterogenic, varied in age (21–60 years of age), dietary and hygienic habits as well as oral cavity status. The above-mentioned initial measurements (before the application of study conditions: freeze-dried diet, isolation, oral hygiene regimen) were considered the control, and final measurements (after the study condition impact) were considered the test group.

To ensure exclusion of numerous environmental variables, rigorous isolation conditions were incorporated and tested in all analog astronaut missions at the LunAres Research Station, including:

- Complete lack of access to sunlight (the time of day was simulated with artificial lighting);
- Strict prohibition to leave the facility for the entire duration of the mission (14 days);
- Limited contact with the outside world barring absolute emergency (only scheduled contact with mission ground control);
- Limited amount of water for personal hygiene;
- Limited amount of space and no privacy;
- Obligatory diet composed of freeze-dried food only.

All participants were subjected to conditions and strict adherence to the rules was mandatory. During the study cumulatively, subjects were exposed to conditions of 434 persons/days.

31 AA × 14 days = 434 person/days(1)

The freeze-dried diet, obligatory for all AA, included 5 meals a day:

1. Breakfast at 8:00;
2. 2nd breakfast at 11:00;
3. Lunch at 13:00;
4. Dessert/snack at 16:00;
5. Dinner at 19:30.

Each of the participants could choose their meals from a provided list (Appendix A) beforehand and during the analog mission had to adhere to predetermined choices and meal preparation instructions, especially in terms of water additions. Possible snacking aside from the schedule was allowed unless it could impact the eating routine. All analog astronauts also need to drink minimum 2 liters of bottled water a day, except for water added to prepare meals which on average was approximately 1000 mL per day (depending on the meal selected, it ranged between 550–1900 mL per day per analog astronaut). In case of warm beverages, the amount was not restricted but could not impact water consumption. Only herbal (mint, rosehip, and chamomile) or fruit (raspberry) infusion was allowed—no coffee nor tea (neither green or black)—to not disturb the regular sleeping patterns of participants with caffeine or theine.

**Figure 4:**
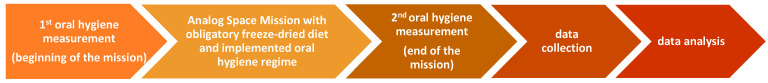
Methodology of the conducted research—single mission.

Measurements were taken at the beginning and at the end of each mission so as to not to interfere with the strict isolation conditions imposed during analog immersion (Figure 4).

The control group of the study consisted of the initial measurement of the subjects, and the test group comprised of the final measurements. In the study, 31 AA (18 women, 13 men) from 15 countries took part, whose ages varied from 21 to 60 years of age (Table 1).

Each of the analog astronauts received the same set of dental products for oral hygiene and was trained in various techniques for effective plaque removal according to the iTOP concept. Every set consisted of the Curaprox ultra-soft 5460 manual toothbrush or the Curaprox smart manual toothbrush—depending on the participants’ oral cavity size—the Curaprox single 1006 manual single-tufted toothbrush, 2 Curaprox Enzycal 950 toothpastes (15 mL), 2 sets of Curaprox interdental brushes in 5 different sizes and 2 small packs of Curaprox waxed dental floss (all mentioned products were produced by CURADEN, Switzerland).

iTOP (individually trained oral prophylaxis) is an original concept of individual training in the field of oral prophylaxis and hygiene, which combines theoretical knowledge and practical training in both manual and motivational skills, developed by the company, Curaden. It enables the implementation of the acquired knowledge in everyday dental routine in order to help patients maintain healthy teeth and periodontium for as long as possible. The research team decided to implement iTOP principals into a oral hygiene regimen to ensure proper use of the provided dental products.

The oral hygiene instructions, to which analog astronauts had to strictly adhere for whole duration of the study (14 days), consisted of:

1. Toothbrushing twice a day (at 8:00 and at 22:00) with the provided multi-tufted toothbrush of choice for a duration of at least 2 min with use of the Bass Technique.
2. According to personal needs (determined by research team), the use of the single-tufted toothbrush, employing the Bass Technique, is complementary to the brushing routine twice a day.
3. Complete interdental cleaning procedure once a day (at 22:00, after toothbrushing) with the use of interdental brushes or floss (in case of crowding) for a duration of at least 2 min.

Four dental indicators were selected and used in the study: the Proximal Plaque Index (API), simplified Oral Hygiene Index (sOHI), Plaque Index by Silness and Loe (PI) and Gingival Bleeding Index by Ainamo and Bay (GBI), due to their high prevalence in oral hygiene and gingival inflammation research.

- API—Proximal Plaque Index—determines the percentage of interdental spaces with plaque accumulation present in relation to all existing interdental spaces and allows one to assess the hygiene in the interdental spaces.
- sOHI—simplified Oral Hygiene Index—determines the ratio of surfaces covered with dental plaque and calculus on selected teeth; the cumulative plaque and calculus score allows one to evaluate the overall oral hygiene.
- PI—Plaque Index by Silness and Loe—determines the thickness of the plaque along the edge of the teeth gingiva in relation to all the cervical area present and allows one to assess the hygiene in the cervical area.
- GBI—Gingival Bleeding Index by Ainamo and Bay (1975)—determines the percentage of the gingival sulcus with bleeding during probing in relation to all examined gingival sulcus present in the area; it enables one to capture gingival inflammation and the early stages of periodontal disease.

The initial oral status of participants showed on average: API scores—33.73%; sOHI scores—0.34; PI—0.29 and GBI—11.59% (Table 1).

**Figure 5:**
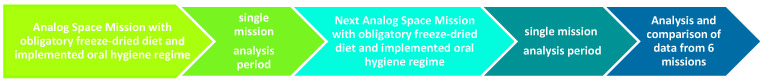
Methodology of data collection and analysis.

Each of the analog astronauts (AA) was assigned a code: AA01–AA31, which allowed for the anonymity of the respondents. Initial and final measurements were assigned the same numbers: 1, 2 and, during each of them, the values of all indicators (API, sOHI, PI, and GBI) were determined. All individual results for each of the analog astronauts were recorded and analyzed (Figure 5).

The differences in the results between the first and second measurements of all participants of 6 analog missions were compared and the statistical significance was calculated. The statistical analysis of the normal distribution data was performed using the Student’s t-test and of the data not following normal distribution was performed using the Mann–Whitney U test.

## 3. Results

Due to the individuality of the results in the respondents, the relationships were considered separately for each of the indicators, and the trends were analyzed separately for each AA and as a whole for the entire group tested.

### 3.1. Approximal Plaque Index

The API values determine the percentage of interdental spaces with present plaque accumulation in relation to all interdental spaces existing in a persons’ oral cavity and allows one to assess the hygiene within the interdental spaces by classifying the test person into one of four categories:

1. 0–24% → optimal hygiene;
2. 25–39% → reasonably good hygiene;
3. 40–69% → average hygiene, requiring improvement;
4. 70– 100% → insufficient hygiene.

**Figure 6:**
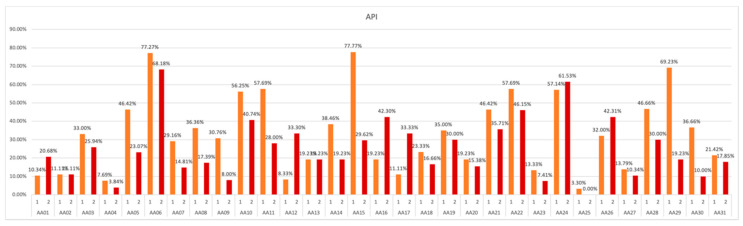
API values obtained in subsequent measurements over the course of study.

Achieving a lower score or category is equivalent to hygiene improvement and obtaining a higher number or category is equivalent to hygiene deterioration. If the hygiene improves during the experiment (i.e., reaches lower values) or does not deteriorate, this demonstrates that the oral hygiene technique was performed correctly (Figure 6).

On the initial measurement (1), we can observe optimal or good hygiene scores in the 21 out of 31 participants (67.74%), which, from a clinical point of view, do not require significant improvement. The remaining 10 analog astronauts (32.26%) obtained API results classifying them into the categories requiring significant hygiene improvement. Category 3 was assigned to 22.58% of participants and category 4 to 9.67%. On the final measurement 17 subjects were classified into optimal or good hygiene groups (80.64%) and only 6 astronauts were in persisting need of hygiene improvement (19.35%), none of which were in category 4.

Due to the clinical significance and application of the indicator as well as the wide spectrum of results obtained from the patients, the interpretation of the results should be carried out with the division of patients into categories and their potential change should be traced.

**Figure 7:**
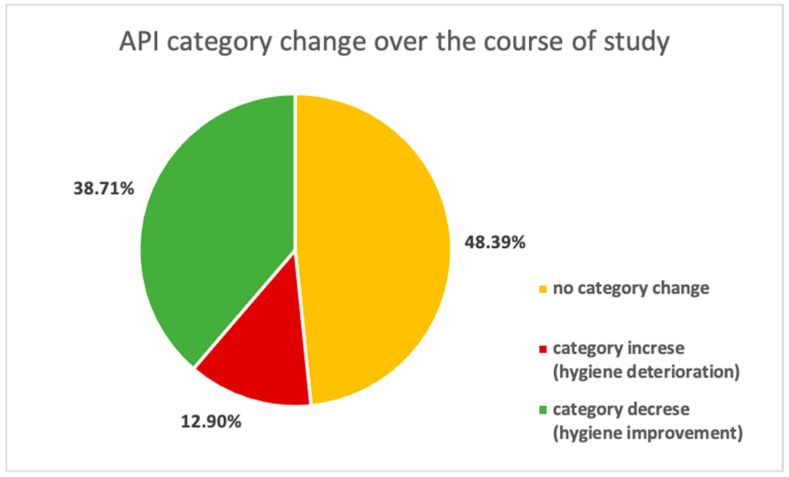
Participants’ potential API category change over the course of study.

The data should also be analyzed in terms of the subjects’ assigned initial and final API category, as achieved scores can be in the borderline category and the participant’s significant improvement/deterioration in hygiene can be omitted (Figure 7).

Tracing the change in the API category over the course of the study, we can observe notable deterioration (category increase) occurring only in 12.90% of participants and significant hygiene improvement (category decrease) in 38.71% of AAs.

### 3.2. Simplified Oral Hygiene Index

The sOHI indicator assesses the amount of plaque and calculus on the tooth surface. The measurement of the two components of plaque and calculus was completed on six teeth on different surfaces, including the facial side of three maxillary teeth, the lingual side of the two posterior mandibular teeth and the labial side of one anterior mandibular tooth. The indicator determines the ratio of the surfaces covered with dental plaque and calculus of the selected teeth to all selected surfaces. The cumulative plaque and calculus score allows one to assess the oral hygiene by classifying the results into three groups:

1. 0–2 → good hygiene;
2. 2.1–4 → sufficient hygiene;
3. 4.1–6 → poor hygiene.

**Figure 8:**
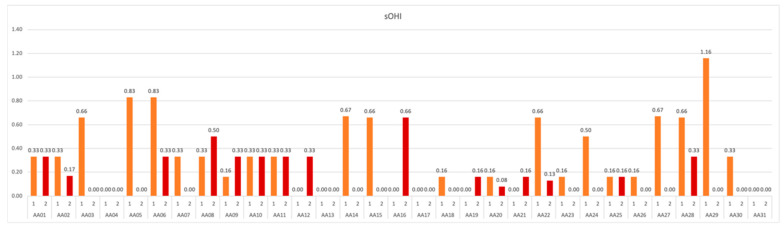
sOHI values obtained in subsequent measurements over the course of study.

The presented data demonstrate that, in 25.81% of the participants, the hygiene was kept at a constant good level, and 54.84% improved over the duration of experiment, where slight worsening of oral hygiene was observed in 19.35% of analog astronauts (Figure 8).

### 3.3. Plaque Index by Silness and Loe

The PI (by Silness and Loe) determines the thickness of the plaque along the edge of the teeth’ gingiva in relation to all cervical area present. The measurement of the state of oral hygiene by the Silness–Löe plaque index is based on recording both soft debris and mineralized deposits on the selected teeth. Each of the four surfaces of the teeth (buccal, lingual, mesial and distal) is given a score from 0–3. The scores from the four areas of the tooth are added and divided by four in order to give the plaque index for the tooth; the procedure is repeated for all selected teeth, and the cumulative plaque index of all teeth is divided into the number of examined teeth. This indicator allows one to assess the hygiene in the cervical area by classifying subjects into three groups:

1. 0–2 → good hygiene;
2. 2.1–4 → sufficient hygiene;
3. 4.1–6 → poor hygiene.

**Figure 9:**
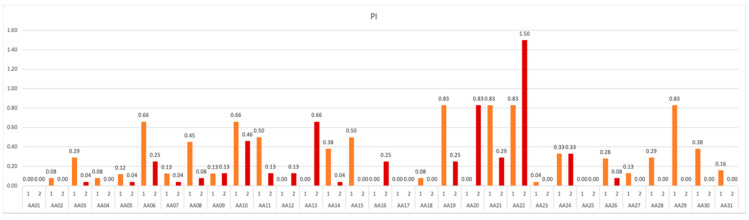
PI values obtained in subsequent measurements over the course of study.

The data show that in 16.13% of the subjects the hygiene was maintained at a constant good level throughout the experiment, and in 67.74% it improved slightly. A small deterioration of the PI score was observed only in 5 out of 31 participants (Figure 9).

### 3.4. Gingival Bleeding Index by Ainamo and Bay (1975)

The GBI—Gingival Bleeding Index by Ainamo and Bay (1975) determines the percentage of the gingival sulcus with bleeding during probing in relation to all examined gingival sulcus area present. Measurement is performed through gentle probing of the orifice of the gingival crevice. If bleeding occurs within 10 s a positive finding is recorded, and if no bleeding occurs, a negative result is noted.

This indicator enables the capture of gingival inflammation and the early stages of periodontal disease, classifying the results obtained from the examined person into four categories:

1. 0–10% → clinically healthy periodontium;
2. 11–29% → localized, mild gingival inflammation;
3. 30–49% → generalized moderate gingival inflammation;
4. 50–100% → severe and generalized gingival inflammation.

**Figure 10:**
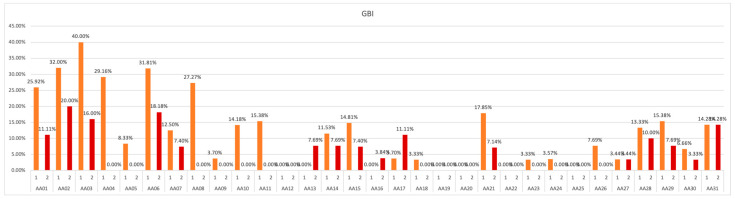
GBI values obtained in subsequent measurements over the course of study.

If, during the analog space mission, a decreasing number of gingival bleeding sides is observed, it represents the reduction of gingival inflammation and the improvement of the periodontium status. This demonstrates the correctness of the oral hygiene techniques. The collected data should be analyzed in terms of a possible increase or decrease, but also considering the assigned GBI category, which is a significant improvement/deterioration of the periodontal health, i.e., when the subject is assigned a lower/higher GBI category, respectively (Figure 10).

The collected data demonstrate that, in 67.74% of participants of the study, there was a decrease in the number of bleeding sites, confirming an improvement of periodontal health. Maintenance of a constant good level throughout the experiment can be observed in 22.58% of the subjects and a small deterioration of the GBI score was noted only in three (out of 31) participants during six analog missions.

Due to the clinical significance and application of the indicator as well as the wide spectrum of results obtained from the patients, the interpretation of the results should be carried out with the division of patients into categories, and their potential change should be traced.

**Figure 11:**
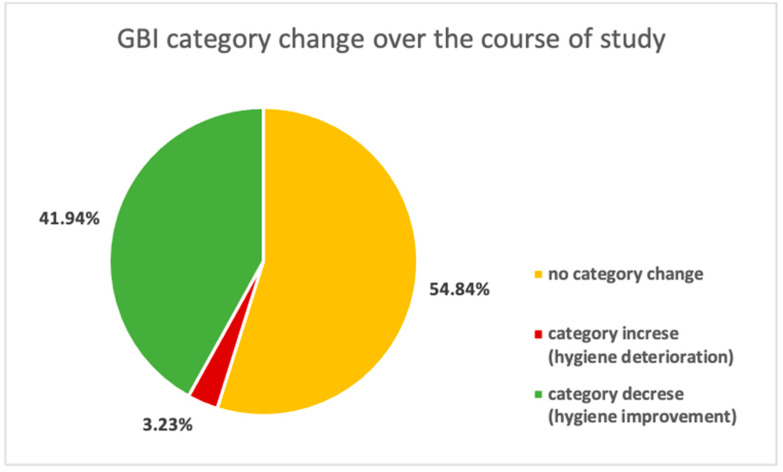
Participants’ potential GBI category change over the course of study.

The data should be analyzed in terms of the subjects’ assigned initial and final GBI category, as achieved scores can be on the category borderline and participant significant hygiene improvement/deterioration can be omitted (Figure 11).

It is also worth noting the clinical significance of the indicator and the potentially significant improvement/deterioration of the gingival status—assigning the lower/higher GBI category, respectively—over the course of the experiment. Tracing the GBI category change over the course of study, we can observe that notable deterioration (category increase) occurred only in 3.23% of participants and significant hygiene improvement (category decrease) in 41.94% of AAs.

### 3.5. Summary and Analysis of Collected Data

**Figure 12:**
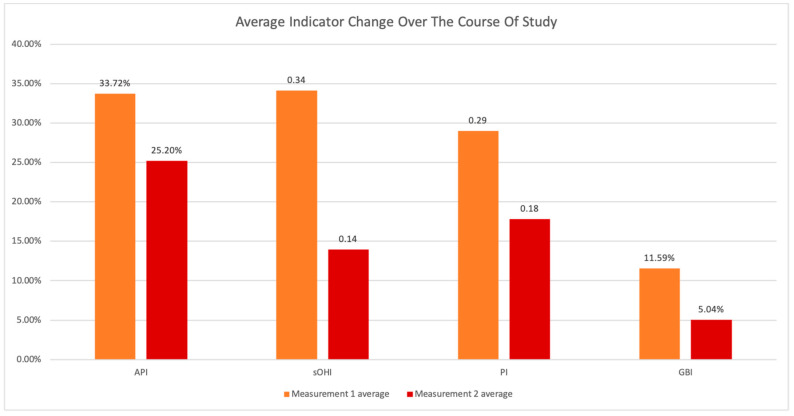
Average indicator change over the course of study.

Individual AA scores were comprehensively analyzed in relation to all collected data and overall average results for initial and final measurements were calculated to observe the average change of particular indicators throughout the experiment (Figure 12).

All data were statistically analyzed in STATISCA 13.3 software and considered statistically significant for *p*-value equal to or less than 0.05. This value proves the correctness of the observed dependencies (Table 2).

Measurements of sOHI, PI and GBI indices demonstrated statistically significant differences (sOHI *p*-value: 0.0068; PI *p*-value: 0.0345; GBI *p*-value: 0.0149), establishing that implemented a oral hygiene regimen with the use of appropriate products and techniques can decrease dental plaque accumulation and gingival inflammation.

The API measurements exhibited a strong downward trend (API *p*-value: 0.0782), which could indicate a decrease in the amount of interdental plaque in the studied analog astronauts, as a result of the applied oral hygiene procedures based on appropriate instruments and techniques.

## 4. Discussion

Research demonstrates that being in isolation causes stress, malaise and depression and may disrupt the daily routine [21]. Maintaining it for a longer period may lead to serious health disorders [22]. These dependencies may lead to loneliness, social isolation [23] and reduced quality of life, which is, unfortunately, often observed more and more among the elderly, who are deprived of contact with other people [24]. Another example of a group struggling with such problems is people in quarantine, in isolation or in extreme space conditions, such as astronauts. In addition to the negative effects on mental health, studies show serious physical consequences of long-term social isolation [24].

In terms of dentistry, isolation can affect the oral hygiene of patients, which can lead to an increased risk of tooth decay and gingivitis. The research clearly demonstrates that the improvement of dental hygiene has a direct positive effect on the periodontal health and contributes to the reduction of the risk of caries [25]. Consumption of starchy staple foods and fresh fruit is associated with low levels of dental caries [26], but because of products’ sophisticated freeze-drying process, of drying after vacuum freezing [9], the food provided to the astronauts does not possess the same qualities. Lyophilized food has a stickier consistency and tends to adhere better to smooth surfaces. As a result, astronauts are much more prone to increased caries and gingival inflammation risks. A prolonged lyophilized diet also has a negative effect on the composition or amount of saliva, due to the dehydration of the organism, which can even lead to xerostomia [18]. The consequences of dry mouth disease may be dental caries, dysgeusia, soreness of the oral mucosa and oral candidiasis [27]. If oral hygiene is neglected in the environment of increased mouth dryness, such as during isolation or daily routine disturbance, it may have a negative effect and progress rapidly [27].

People in extreme conditions, such as astronauts, are often subjected to very high stress and are isolated from others. This makes them a good research group for the relationship between isolation, dental hygiene and a freeze-dried diet. In this study, it was observed that even in unfavorable dietary and stressful isolation conditions, the mentioned trends were confirmed, and that they do not determine the persons’ oral hygiene status as well as periodontal health. In conditions of appropriate oral hygiene technique and establishing oral hygiene daily routine, improvement in oral health and dental hygiene can be observed within only 14 days. The lack of statistical significance for all indicators is probably due to the wide spectrum of API scores within a small tested group, whilst the literature suggests that this hygiene indicator used in larger studies is very reliable and often demonstrates hygiene improvement or deterioration [28].

The observations made by the research team are directly relevant to everyday life, especially for people staying in quarantine or enduring prolonged isolation conditions. Specially designed oral hygiene regimen could be also implemented in the preparation process for people planning endeavors, such as space travel, mountain climbing or survival trips. Especially for astronauts, the preparation process often takes a very long time, during which proper hygiene habits could be developed and sustained throughout the duration of the mission, which could minimize the risk of caries or gingivitis occurrence.

It has been reported that some organic and environmentally friendly solutions, such as green tea, have been tested and demonstrated a promising beneficial impact on periodontal tissues [29], but due to the strict isolation and dietary regulations of this study (drinking green tea was not allowed for participants), they were not taken under consideration.

Other studies suggest the use of novel organic solutions, such as ozonated olive oil, as mouthwash for patients with periodontal problems [30], but most likely in non-severe cases, well-designed hygiene instructions and adherence to strict hygiene regimen can bring positive effect without the use of any mouthwash, to which analog astronauts did not have access to throughout the course of the study. In case of the presence of participants with more severe periodontal problems, this novel solution can be considered in future studies.

Codispoti et al. suggest that solutions to aid patients with more severe periodontal problems can be technologies based on exosomal activity (NANOBIOME) [31,32], which could help, especially in remote conditions or prolonged isolation. People returning from an environment where sometimes oral hygiene is not a top priority, could use such novel approaches to improve their poor oral state after neglecting their hygiene. Despite this unique perspective, the research team concluded from the gathered data (where severe cases of periodontal issues were not present) that adherence to instructions and strict hygiene regimen can be sufficient in most cases.

Due to the fact that analog space research is not yet mainstream and that this field still needs a lot of expansion, most studies conducted during analog missions are innovative and pioneering. In addition, because of the low number of facilities and their availability to researchers from all science spectra, some research has never been conducted before in the aforementioned extreme conditions and on this particular test group. The constant development of research and its interdisciplinary nature as well as the uniqueness of the studied conditions makes this difficult to discuss with other researchers, due to the limited number of publications about the subject matter.

### Limitations

The main limitations of the study were the small and ethnically heterogenic group of study participants, who varied in age (21–60 years of age), dietary and hygienic habits as well as oral cavity status. The only exclusion factors for study participation were: lack of good state of overall health and presence of serious gastric problems, which could restrict the analog astronauts from consuming the freeze-dried diet.

## 5. Conclusions

(1) The study demonstrates that a freeze-dried diet in isolation conditions exclusively appears to not have a negative effect on oral hygiene and gingival status.
(2) The most probable cause of plaque accumulation and gingival inflammation, intensified by sticky consistency of freeze-dried food, seems to be the neglect of oral hygiene habits.
(3) Individual oral hygiene training (iOHT) ensures correct technique performance and maintaining regular hygiene habits during isolation. iOHT should be incorporated into a preparation protocol for analog space missions and other extreme isolation endeavors.
(4) Research should be continued at the LunAres Research Station to increase the sample size.

## Figures and Tables

**Table 1:** Patient and dental material information at the beginning of study.

Patient data		Medium	Range
	Age	31	21–60
Dental data	DMFT	7.516	0–17
	API	33.73%	3.30–77.70%
	sOHI	0.34	0–1.16
	PI	0.29	0–0.83
	GBI	11.59%	0.00–40.00%

**Table 2:** Statistical significance of individual examined indicators.

**Oral Hygiene/Gingival Inflammation Indicator**	***p*–Value**
Approximal Plaque Index (API)	0.0782
Simplified Oral Hygiene Index (sOHI)	0.0068
Plaque Index by Silness and Loe (PI)	0.0345
Gingival Bleeding Index by Ainamo and Bay (GBI)	0.0149

## Data Availability

Not applicable.

## Appendix A

Dietary information of the analog space mission.

Each of the participants could choose their meals (breakfast, second breakfast, lunch, dessert, and dinner) from the following list and during the analog mission had to adhere to predetermined choices and meal preparation instructions.

All freeze-dried products were produced by the company, LYOFOOD Sp. z o. o. (Poland).

**Table:**

Type of Meal	Dish Name	Net Weight (g)	Amount of Added Water (mL)	Ingredients
Breakfast	Organic millet porridge with raspberries and aronian powder	100	200	organic millet 72%, organic coconut milk 19%, organic agave syrup 6%, organic raspberry crumble 2%, organic aronia powder 1%
	Organic poridge with apple, cranberries, cinamon and chia seeds	70	140	bio gluten-free oats 54%, bio coconut milk 37%, bio agave syrup, bio apple cubes 2%, bio chia seeds, bio cranberry slices, bio cinnamon powder
	Coconut porridge with blueberries, figs and chia seeds	100	200	gluten-free oats 53%, organic coconut milk 37%, figs slices 4%, organic agave syrup 4%, organic chia seeds, blueberries whole
	Mexican scrambled eggs	75	195	egg mix (eggs, citric acid) 39%, red bell pepper 14%, onion 14%, sweet corn, red bean 12%, cheese (sheep’s milk, salt, rennet), olive oil, coriander leaves, chili, garlic, salt
2nd breakfast	Fruity dream	30	0	freeze-dried strawberries 33%, apple 33%, sour cherries 33%
	Wild berry mix	30	0	freeze-dried raspberries 33%, blueberries 33%, blackberries 33%
	Exotic pleasure	30	0	freeze-dried tangerines 25%, kiwi 25%, banana 25%, pineapple 25%
	Strawberries	20	0	freeze-dried strawberries 100%
	Banana	30	0	freeze-dried banana 100%
	Apple	30	0	freeze-dried apple 100%
	Cherry	30	0	freeze-dried sour cherry 100%
	Porridge apple, cinamon, cranberry	55	155	Ingredients: bio gluten-free oats 54%, bio coconut milk 37%, bio agave syrup, bio apple cubes 2%, bio chia seeds, bio cranberry slices, bio cinnamon powder
	Cocoa granola with strawberries	65	155	gluten-free oats, agave syrup, strawberries, cocoa
Lunch/Dinner	Organic gazpacho	25	225	bio tomatoes (tomatoes, tomato juice) 57%, bio cucumber 33%, bio red bell pepper 5%, bio onion, bio olive oil, salt, bio apple vinegar, bio chili
	Cream of tomato and pepper soup	37	333	26% tomato pulp (tomatoes, salt, citric acid), 22% sour cream, 22% rice, 13% red bell pepper, onion, 7% tomato paste, salt sugar, spices
	Cream of leek & onion soup	37	333	26% onion, 26% leek, 17% potatoes, 14% chickpea, 7% cheese pecorino romano (PDO), 5% sour cream, chive, salt, spices
	Cream of mushroom soup with gorgonzola and pasta	65	305	34% pasta (durum wheat semolina), 23% champignons, onion, carrot, 7% mushrooms (bay bolete, boletus), sour cream, 3% dried mushrooms (boletus, bay bolete), 3% cheese gorgonzola (PDO), parsley leaves, salt
	Cream of broccoli and spinach soup	60	310	38% broccoli, 33% spinach, onion 7% mozzarella cheese, 6% sour cream, 4% pumpkin seeds, 2% cheddar cheese, 1% spices (contains celery), salt
	Goulash soup	80	420	pork meal 20%, potatoes, red bell pepper, bean 15%, wheat flour, carrot, onion, parsley root 5%, tomato paste, salt, canola oil, spices
	Organic chili sin carne bistro	45	165	polenta, red bell pepper, tomatoes, red beans, onion, green olives, canola oil, spices, chilli, salt
	Lentile dhal bistro	55	155	millet, red lentils, coconut milk, tomatoes, onion, green lentils, olive oil, spices (curcuma, cumin, coriander, ginger, cinnamon), chili, salt
	Moroccan stew	45	165	polenta, onion, zucchini, chickpeas, carrot, red bell pepper, olive oil, salt
	Chana masala bistro	60	150	rice, coconut milk, tomatoes, onion, pumpkin, chickpea, olive oil, spices (curcuma, cumin, coriander, chili), potato flour, garlic, ginger, lime juice, coriander leaves, parsley leaves, salt
	Stew with pearl barley	112	388	pork meal 36% (cubes), pearl barley 25%, carrot 5%, red bell pepper 5%, green peas 5%, parsley root 5%, onion 5%, wheat flour, canola oil. salt, spices
	Penne alla bolognese	128	372	pasta 37% (durum wheat semolina), 18% tomato pulp (tomatoes, salt, citric acid), ground pork meat 8%, ground beef meat 8%, carrot 4%, onion 4%, tomato paste 4%, wheat flour, salt, canola oil, spices
	Barley lentils risotto with avocado mousse	128	372	red bell pepper 41%, green lentils 14%, pearl barley 12%, leek, avocado 5%, pumpkin seeds, lemon juice, parsley, olive oil, salt, spices, herbs
	Pork loin in green pepper	79	291	pork loin 35% (cubes), potatoes 26%, green beans, onion, wheat flour, green peppercorn 2%, canola oil, salt, spices
	Pork loin in green pepper	107	393	pork loin 35% (cubes), potatoes 26%, green beans, onion, wheat flour, green peppercorn 2%, canola oil, salt, spices
	Pork loin in dill	77	293	pork loin 35% (cubes), potatoes 26%, carrot, parsley root, leek, onion, broccoli, cauliflower, wheat flour, canola oil, salt, spices, dill 0.03%
	Pork loin in dill	104	396	pork loin 35% (cubes), potatoes 26%, carrot, parsley root, leek, onion, broccoli, cauliflower, wheat flour, canola oil, salt, spices, dill 0.03%
	Organic chili sin carne	70	300	bio polenta 28%, bio red bell pepper 19%, bio tomatoes (tomatoes, tomato juice), bio red bean 15%, bio onion, bio green olives, bio canola oil, bio spices, salt, bio chili 0.04%
	Mexican dish	94	276	chicken breast fillet 35% (cubes), rice 26%, red bean 11%, red bell pepper 4%, sweet corn 4%, onion, wheat flour, tomato paste, canola oil, salt, spices
	Mexican dish	126	374	chicken breast fillet 35% (cubes), rice 26%, red bean 11%, red bell pepper 4%, sweet corn 4%, onion, wheat flour, tomato paste, canola oil, salt, spices
	Bigos	80	420	ssauerkraut 59% (sauerkraut, carrot, salt), pork meat 17% (cubes), 6% chicken fillet (cubes), champignon mushrooms, tomato paste, canola oil, dried boletus mushrooms, smoked plums, spices
	Farfalle with gorgonzola and spinach sauce	94	276	spinach 44%, pasta 27% (durum wheat semolina), onion, sour cream, gorgonzola 5%, almonds 2%, salt garlic, butter
	Farfalle with gorgonzola and spinach sauce	126	374	spinach 44%, pasta 27% (durum wheat semolina), onion, sour cream, gorgonzola 5%, almonds 2%, salt garlic, butter
	Five spice chicken	82	288	rice 33%, chicken breast fillet 28% (cubes), vegetables in varying proportions 23%, (mung bean sprouts, red bell pepper, red onion, jelly ear mushrooms, bamboo shoots, carrot, leek, onion), potato flour, salt, soy sauce (soy beans, salt, wheat flour), spices
	Five spice chicken	100	400	rice 33%, chicken breast fillet 28% (cubes), vegetables in varying proportions 23%, (mung bean sprouts, red bell pepper, red onion, jelly ear mushrooms, bamboo shoots, carrot, leek, onion), potato flour, salt, soy sauce (soy beans, salt, wheat flour), spices
	Chicken tikka masala	128	372	rice 26%, tomato pulp 23% (tomatoes, salt, citric acid), chicken breast fillet (strips) 21%, greek yoghurt 20%, coconut milk 4%, almonds 2%, canola oil, salt, spices (contains mustard), sugar
	Strogonof	113	257	noodles 41% (durum wheat semolina), beef 33% (strips), champignons 7%, onion 4%, red bell pepper, wheat flour, pickled cucumber, mustard (mustard seeds, vinegar, salt, suger, turmeric), tomato paste, canola oil, salt, paprika, pepper, spices
	Nettle curry	110	390	rice 30%, coconut milk 25%, sugar peas, carrot, pumpkin, broccoli, zucchini, coriander leaves, nettle 1%, lime juice 1%, lemon grass, salt, green chili 0.4%, spices
	Beef strogonof	152	348	noodles 41% (durum wheat semolina), beef 33% (strips), champignons 7%, onion 4%, red bell pepper, wheat flour, pickled cucumber, mustard (mustard seeds, vinegar, salt, suger, turmeric), tomato paste, canola oil, salt, paprika, pepper, spices
	Organic lentil dal with millet	97	270	bio millet 37%, bio tomatoes (tomatoes, tomato juice)14%, bio red lentils 12%, bio coconut milk 12%, bio onion, bio green lentils 5%, bio olive oil, salt, spices, cumin, curcuma, ginger
Snack/Dessert	Catalonian cream	65	135	77% milk origin: Poland, 10% egg yolks, 6% sugar, potato flour, orange peel, cinnamon
	Apple crumble	70	70	wheat flour 39%, butter 29%, sugar, apple 13%, cinnamon
	Chocolate pudding	65	65	milk 62%, chocolate 18%(cocoa paste, cane sugar, cocoa butter), sugar, egg mix 6%(eggs, citric acid), potato flour
	Red smoothie	42	458	strawberries 29%, banana 29%, peach 21%, blackcurrant 14%, cranberries 7%
	Green smoothie	42	458	apple 35%, kiwi 35%, pineapple 21%, spinach 4%, nettle 4%, ginger
	Red vitamin drink	51	449	strawberry 53%, blackcurrant 35%, beetroot 12%
